# Ten-Year Experience with Transapical and Direct Transaortic Transcatheter Aortic Valve Replacement to Address Patients with Aortic Stenosis and Peripheral Vascular Disease

**DOI:** 10.3390/jcdd9120422

**Published:** 2022-11-28

**Authors:** Enrico Ferrari, Alberto Pozzoli, Catherine Klersy, Francesca Toto, Tiziano Torre, Tiziano Cassina, Giovanni Pedrazzini, Stefanos Demertzis

**Affiliations:** 1 Cardiac Surgery, Cardiocentro Ticino Institute, Ente Ospedaliero Cantonale, 6900 Lugano, Switzerland; 2 Biomedical Faculty, University of Italian Switzerland (USI), 6900 Lugano, Switzerland; 3 School of Medicine, University of Zurich, 8006 Zurich, Switzerland; 4 Clinical Epidemiology & Biostatistics, Fondazione IRCCS Policlinico San Matteo, 27100 Pavia, Italy; 5 Cardiac Anaesthesia, Cardiocentro Ticino Institute, Ente Ospedaliero Cantonale, 6900 Lugano, Switzerland; 6 Cardiology, Cardiocentro Ticino Institute, Ente Ospedaliero Cantonale, 6900 Lugano, Switzerland

**Keywords:** transcatheter aortic valve replacement, aortic valve stenosis, direct transaortic access, transapical access

## Abstract

**Objective**: Transcatheter aortic valve replacement (TAVR) through alternative access routes is indicated in patients with severe aortic valve stenosis and diseased peripheral arteries. We analysed and compared the outcome of patients undergoing transapical (TA) and direct transaortic (TAO) TAVR procedures. **Methods**: Preoperative characteristics, procedural details, and thirty-day outcome of patients undergoing transapical (TA-TAVR group) and direct transaortic (TAO-TAVR group) TAVR procedures were prospectively collected and retrospectively analysed. **Results**: From March 2012 to March 2022, 81 TA and 82 TAO-TAVR (total: 163 cases) were performed with balloon-expanding (*n* = 120; 73.6%) and self-expandable (*n* = 43; 26.4%) valves. The mean age was 79.7 ± 6.2 and 81.9 ± 6.7 years for the TA- and TAO-TAVR groups, respectively (*p* = 0.032). Females were more represented in the TAO-TAVR group (56% vs. 32%; *p* = 0.003) while TA-TAVR patients showed a higher prevalence of previous vascular surgery (20% vs. 6%; *p* = 0.01), previous cardiac surgery (51% vs. 3.6%; *p* < 0.001), and porcelain aorta (22% vs. 5%; *p* = 0.001). The mean ejection fraction was 49.0 ± 14.6% (TA) and 53.5 ± 12.2% (TAO) (*p* = 0.035) while mean gradients were 35.6 ± 13.2 mmHg (TA) and 40.4 ± 16.1 mmHg (TAO) (*p* = 0.045). The median EuroSCORE-II was 5.0% (IQR: 3.0–11.0) and 3.9% (IQR: 2.5–5.4) for the TA- and TAO-TAVR groups, respectively (*p* = 0.005). The procedural time was shorter for TA procedures (97 min (IQR: 882–118) vs. 102 min (IQR: 88–129); *p* = 0.133). Mortality at day 30 was 6% in both groups (*p* = 1.000); the permanent pacemaker implantation rate was similar (8.6% vs. 9.7%; *p* = 1.000), and hospital stay was shorter for the TAO group (8 days (IQR: 6–11) vs. 10 days (IQR: 7–13); *p* = 0.025). **Conclusions**: Our results show that transapical and direct transaortic TAVR in high-risk patients with diseased peripheral arteries provide satisfactory clinical results with similar thirty-day outcomes.

## 1. Introduction

Aortic valve stenosis is the most common heart valve disease in adults and standard surgical replacement remains the treatment of choice [1,2,3]. However, elderly patients, patients at risk for surgery, patients undergoing reinterventions, and, more recently, also patients with an intermediate risk profile can be successfully treated with a transcatheter aortic valve replacement (TAVR) performed through a transfemoral access or alternative access routes [4,5,6,7,8,9]. The transfemoral access (TF) is a less invasive access site, and candidates for TAVR are, at first, screened for the quality and the size of the aorta and ileo-femoral arteries using computed tomography scan (CT) images with 3D reconstructions. In cases of severe atherosclerosis, calcifications, vessels with a small diameter, or severe tortuosities, alternative accesses for the TAVR, namely transapical (TA), direct transaortic (TAO), trans-subclavian, and trans-carotid, might be considered. However, there is a lack of evidence of technical or clinical advantages in performing either a TA- or TAO-TAVR in this subgroup of high-risk patients with diseased peripheral arteries who are not eligible for standard TF-TAVR. The present study investigates the hospital results and the thirty-day outcomes of patients undergoing TA- and TAO-TAVR procedures with a particular focus on moderate/severe paravalvular leaks (PVL) and permanent pacemaker implantation rate.

## 2. Methods

### 2.1. Study Design

This is a retrospective single-centre observational study included all consecutive patients undergoing transapical (TA-TAVR group) or direct transaortic (TAO-TAVR group) transcatheter aortic valve replacements during a period of 10 years (March 2012–March 2022). Patients with symptomatic aortic valve stenosis and at moderate or high risk for surgery were screened for TAVR by our institutional Heart Team. Patients presenting with unsuitable peripheral arteries at 3D CT-scan analysis did not undergo standard transfemoral TAVR and were treated using alternative access routes. Clinical and technical details were prospectively collected and retrospectively analysed, and then patients were divided into two groups: the TA-TAVR group and the TAO-TAVR group. Postoperative complications were collected following the international VARC-3 guidelines and the thirty-day outcomes of both groups were compared [10]. All patients signed the informed consent for the transcatheter valve procedure and the use of anonymized clinical data for clinical research and quality control purposes. All TAVR patients were also included in the nationwide Swiss TAVI-Registry database (registered at clinicaltrials.gov, n. NCT01368250) and approved by the Ethics Committee (number 056/11). The present investigation abides by the principles outlined in the Declaration of Helsinki (Ethical Principles for Medical Research Involving Human Subjects) adopted by the 18th WMA General Assembly in Helsinki, Finland, June 1964.

### 2.2. Patients Selection and Clinical Management

Elderly patients with severe symptomatic aortic stenosis who were not eligible for a safe TF-TAVR underwent TA- or TAO-TAVR procedures. Femoral arteries smaller than 6 mm diameter (unsuitable for 18-Fr introducers) or vessels presenting severe concentric annular calcifications, complex tortuosities, aortic or iliac aneurysms with intravascular thrombi (in particular, in the aortic arch), or patients with aortic endografts or iliac stents were considered at risk for vascular complications and therefore not eligible for the TF-TAVR. The principle of “not forcing the indication for TF” for the patient’s safety was adopted by the Heart Team since the beginning of our activity. The allocation to the TA- or TAO-TAVR group was discussed within the Heart Team: In the absence of a specific contraindication to a specific procedure, the decision was based on a 1:1 ratio in order for the team to maintain the technical skills and expertise of both transcatheter techniques (all TAVR proceedures performed by all Heart Team members with one cardiac surgeon performing all non-TF TAVR). These inclusion criteria never changed over time. The aortic annulus was assessed by cardiac CT scan using the 3-Mensio program system (Pie Medical Imaging, Maastricht, The Netherlands): the diameter, the area, and the perimeter of the annulus were used for valve sizing. All TAVR procedures were performed in a hybrid operating room under fluoroscopic control, general anaesthesia, and periprocedural transoesophageal echocardiographic control. On day 30, all discharged patients underwent a postdischarge clinical evaluation at our outpatient clinic.

### 2.3. TA-TAVR

Patients in this group underwent TAVR through a left anterolateral mini thoracotomy at the fifth intercostal space. The ventricular apex was prepared with two reinforced concentric 3-0 polypropylene purse-string sutures, and temporary epicardial wires were used for the rapid pacing. Implanted devices were the Sapien XT (2012–2013), the Sapien-3 (2014–2018), the Sapien-3 Ultra (2019–2022) (Edwards Lifesciences, Irvine, CA, USA), and the Accurate (Boston Scientific, Marlborough, MA, USA). Uncomplicated patients were extubated in the hybrid room and transferred to the intensive care unit for surveillance. Contraindications for TA-TAVR were a fresh apical thrombus, a severe thorax deformity, or a dilated left ventricle with impaired function.

### 2.4. TAO-TAVR

Patients in this group underwent TAVR through a right anterolateral mini thoracotomy at second intercostal space. The aorta was prepared with two concentric reinforced 3-0 polypropylene purse-string sutures, and the pacemaker wire for rapid pacing was placed intravenously [11]. Implanted devices were the Sapien XT (2012–2013), the Sapien-3 (2014–2018), the Sapien-3 Ultra (2019–2022) (Edwards Lifesciences, Irvine, CA, USA), and the CoreValve (Medtronic, Minneapolis, MN, USA). Uncomplicated patients were extubated and transferred to the intensive care unit for surveillance. Contraindications were heavy calcifications of the ascending aorta and previous cardiac surgery with patent venous grafts.

### 2.5. Statistical Analysis

Statistical analysis was performed using Stata release 17 (StataCorp, College Station, TX, USA). A *p*-value lower than 0.05 was considered statistically significant. All tests are 2-sided. Continuous variables are described as mean ± standard deviation (SD) or median and quartiles (IQR) and compared with the Student’s *t*-test or the Mann–Whitney U test, respectively. Categorical variables are presented as numbers and proportions (%) and a Fisher exact test was used for comparisons. The difference in the rate of 30 days outcomes between TA and TAO approaches was reported together with a 95% confidence interval (95% CI). No multivariable analysis was performed given the low number of events.

## 3. Results

### 3.1. Baseline Characteristics

Baseline characteristics and echocardiographic data of TA- and TAO-TAVR patients are described in Table 1 and Table 2. From March 2012 to March 2022, 81 TA and 82 TAO-TAVR were performed (roughly 20% of all TAVR procedures). The mean age was 79.7 ± 6.2 and 81.9 ± 6.7 years for the TA- and TAO-TAVR groups, respectively (*p* = 0.032), with females less represented in the TA group (32% vs. 56%; *p* = 0.003). With regards to comorbidities, previous vascular surgery (20% vs. 6%; *p* = 0.010) and previous cardiac surgery (51% vs. 3.7%; *p* < 0.001) were significantly more prevalent in the TA-TAVR group. The median EuroSCORE-II was higher in the TA group (5.0% (IQR 3.0–11.0) vs. 3.9% (IQR 2.5–5.4); *p* = 0.005) while the echocardiographic findings showed significantly different mean valve gradients (35.6 ± 13.2 mmHg for TA and 40.4 ± 16.1 mmHg for TAO; *p* = 0.045) and left ventricular ejection fraction (49.0 ± 14.6% for TA and 53.5 ± 12.2% for TAO; *p* = 0.035).

### 3.2. Operative Outcomes

The success rate (defined as the implantation of at least one transcatheter valve in the aortic position with the patient alive at the end of the procedure) was 99%: one patient in the TA-TAVR group had cardiac arrest after the valve deployment and never recovered. Moreover, three patients in the TA group and one in the TAO group had a bailout TAV-in-TAV for the malposition of the first transcatheter valve with consequent moderate-to-severe PVL. Implanted TAVR valve type, mean valve size, and size distribution are listed in Table 3.

Balloon-expandable valves were used in 120 cases (73.6%) and self-expanding valves were used in 43 cases (26.4%). The median procedural time was slightly longer for the transaortic procedures (102 (IQR: 88–129) vs. 97 (IQR: 82–118) minutes; *p* = 0.133). The transapical procedure required less contrast: 125.1 ± 105.5 vs. 174.5 ± 90.7 mL (*p* < 0.001).

### 3.3. 30 Days Outcome

**Figure 1:**
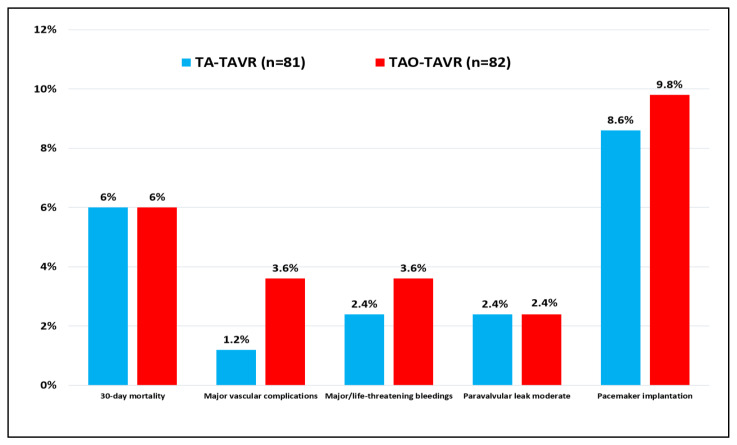
Postoperative complications after TA-TAVR and TAO-TAVR procedures according to VARC-3 definitions.

With regards to the thirty-day mortality and complication rate, variables were collected following the VARC-3 definitions (Table 4; Figure 1). Differences in the outcome and 95% CI are also shown. Hospital mortality was 6% for both groups. No death was observed after discharge from the hospital. Cardiovascular mortality occurred in one TA-TAVR patient and three TAO-TAVR patients. Major vascular complications (1.2% vs. 3.6%; *p* = 0.367) and major bleedings (2.4% vs. 3.6%; *p* = 1.000) occurred more often in the TAO-TAVR group. Neurological complications were never reported while acute kidney injury requiring dialysis occurred in 3 TA (3.7%) and 2 TAO (2.4%) patients. As per the onset of new conduction abnormalities leading to permanent pacemaker implantation, seven devices (8.6%) were implanted in the TA group and eight (9.7%) in the TAO group (*p* = 1.000). The median hospital stay was slightly longer for TA patients: 10 (IQR: 7–13) and 8 (IQR: 6–11) days for TA and TAO, respectively (*p* = 0.025). The predischarge echocardiographic control showed mean gradients of 9.7 ± 4.8 mmHg (TA) and 9.6 ± 3.5 mmHg (TAO) (*p* = 0.830) with moderate PVL detected in two patients (2.4%) for both groups.

## 4. Discussion

Since the beginning of transcatheter aortic valve therapies, the transfemoral and transapical have been the two main access routes, with other alternative access routes being developed over the years. Nowadays, the TF represents the most attractive approach given the lower degree of surgical invasiveness and the faster patient recovery and discharge. However, candidates with diseased peripheral arteries not fulfilling the criteria for a safe and noncomplicated TF-TAVR can be treated with TAVR performed through alternative access routes, but the risk profile of these patients is expected to be higher because the vascular disease is a variable included in the risk scores in use.

In our institution, the TA-TAVR and the TAO-TAVR are the most popular not-transfemoral TAVR procedures performed, and only in a few cases, the trans-subclavian or the trans-carotid are used as alternative access routes. We investigated the thirty-day outcomes of our cohort of TA and TAO patients with symptomatic aortic valve stenosis and peripheral artery disease, and the results are in line with data published in the available literature and comparable between the two groups.

Regarding the thirty-day mortality, we calculated a mean predicted mortality rate of 8.6% for TA cases and 5.6% for TAO cases based on the EuroSCORE-II scoring system, and we observed a hospital mortality rate of 6% for both groups. It is of interest that all patients discharged from the hospital survived the thirty-day follow-up. In a similar, very large multicenter study by Thourani et al., the TA and TAO accesses were compared, and no significant difference in thirty-day mortality was found, even after patient risk adjustment (14% vs. 9%; *p* = 0.283). Notwithstanding, a trend towards a higher one-year survival rate with TAO-TAVR was also documented [12]. Furthermore, in another systematic review and meta-analysis comparing 9619 TA patients with 342 TAO patients, the thirty-day mortality rate did not differ significantly (7.9 vs. 9.7%) [13]. Last, in the observational study by Arai, 289 cases of TAO-TAVR and 42 cases of TA-TAVR were analysed and no significant difference in thirty-day mortality between the two techniques was detected (9% vs. 14%; *p* = 0.283) [14].

Regarding the neurological complications, we did not detect strokes nor TIA in the two cohorts and neurologic protection devices were not used during the procedures. In the systematic review and meta-analysis previously mentioned, a trend toward a lower rate of stroke in the transaortic group was noted (0.9% vs. 2.1%) but without statistical significance [13]. Some differences (though not significant) between the groups are reported when post-TAVR vascular complications are concerned. In particular, vascular complications were more often reported in TAO-TAVR patients (1 vs. 3 cases; *p* = 0.367), where we faced one late aortic annular rupture (heavily calcified annulus), one aortic rupture during the procedure requiring the conversion to a mean sternotomy for repair (dilated and fragile ascending aorta damaged by a self-expanding valve), and one pseudoaneurysm of the femoral artery at the insertion point of the pigtail for angiograms that was surgically treated. Only the aortic annular rupture caused the death of the patient. The TA group only experienced one case of femoral pseudoaneurysm requiring surgery. Major bleedings post-TAVR were equally distributed between the two groups (2.4% and 3.6%, respectively). Therefore, despite the transapical access still representing a challenge in frail patients, the results show a low risk of major vascular complications, and, consequently, a low risk of apical bleeding. Bleeding from the apex requiring a rethoracotomy was never reported in our series, and the two reported rethoracotomies in the TA group were due to diffuse bleedings without open source. The available literature reported overall a few patients (2.1% [95% CI, 0.5% to 4.4%]) who required conversion to open surgical valve replacement. In published TA and TAO cohorts, the major bleedings accounted for 9.4% in the TA group and 8% in the TAO group, which are sensibly higher than our results [13]. New-generation devices with low-profile and small-sized introducer sheaths with small delivery catheters will probably even further decrease the incidence of access-site-related vascular complications and major bleedings in future clinical reports.

With regards to the presence of moderate/severe PVL at discharge, we report a 2.4% in both groups, without severe PVL detected. These data were definitely better than the ones reported in the literature. Data from the TVT Registry demonstrated a more than mild site-reported PVL rate of 5.8% at discharge without apparent differences between the two access routes [12]. The systematic review by Dunne showed moderate or worse PVL at 5% in the TA cohort and 4.1% in the TAO cohort [13]. In the report by Arai, moderate/severe PVL was 5% for TA patients and 7% for the TAO group (*p* = 0.424), slightly favouring the TA patients [14]. Again, our results are favourable if compared with published data. In our experience, we have seen a great improvement in PVL rate after the launch of the last-generation transcatheter valves featuring innovative outer skirts aiming at preventing post-TAVR leaks. It is reasonable to consider that the introduction of new-generation devices will even further reduce the incidence of clinically relevant postoperative leaks, leading to better long-term outcomes, especially when younger patients undergoing TAVR will be concerned.

Last but not least, the need for new permanent pacemaker implantation following TAVR procedures complicated by high-degree conduction abnormalities was similar in both our groups (8.6% for TA and 9.8% for TAO; *p* = 1.000) despite self-expanding valves (known for causing a higher incidence of conduction disturbances) were more often used in the TAO group (24% vs. 47.6%, respectively). These results are interestingly better than data recently reported from the multicentre OPERA-TAVI registry comparing very last-generation, self-expanding and balloon-expandable transcatheter devices [15]. The permanent pacemaker implantation rate was reported as 17.9% for the self-expanding cohort and 10.1% for the balloon-expandable cohort. From this perspective, we can even assume a positive effect of the alternative access site allowing for a more stable and precise higher valve positioning, therefore, limiting the risk of conduction disturbances.

The development of new generation valve devices with low-profile delivery catheters assuring lower incidence of paravalvular leaks, pacemaker implantation rates, and vascular injuries will further improve TAVR safety and efficacy not only during transfemoral procedures but also when alternative access routes are required. This aspect can have a great impact on future reports on TAVR outcomes allowing for the use of these new devices in low-risk and younger patients, regardless of the entry site.

In the end, we should also say that the Heart Team is of paramount importance when patients with artery disease are candidates for a TAVR [16]. The scope of the Heart Team is investigating all potential alternative strategies to safely treat patients without complications and long hospitalizations. In this regard, the TF approach remains the main access route for TAVR, but a well-structured, open discussion about the use of alternative approaches between the members of the team becomes mandatory as soon as the vascular accesses are complex and/or diseased. In our opinion, the dogma of performing the TF-TAVR “whatever it takes” should no longer be pursued in a modern and dynamic Heart Team because we must guarantee the best clinical result for our patients.

This study has some limitations. This is a nonrandomised retrospective observational study describing the thirty-day outcome of selected patients undergoing TA- and TAO-TAVR procedures in one single institution. Our study compared the patients’ characteristics and used the thirty-day mortality as an outcome without a longer follow-up. Other aspects such as the specific valve-related outcomes, including symptom improvement, quality of life, and structural valve deterioration, have not been assessed.

## 5. Conclusions

The present study shows that TA- and TAO-TAVR procedures can be safely performed in high-risk patients by an experienced Heart Team. In order to guarantee safe and reliable clinical results over time, these alternative access routes should be performed on a regular basis.

## Figures and Tables

**Table 1:** Baseline characteristics.

VARIABLES	Overall (*n* = 163)	TA-TAVR (*n* = 81)	TAO-TAVR (*n* = 82)	*p*-Value
Age (years)	80.8 ± 6.5 (range: 57–97)	79.7 ± 6.2 (range: 65–93)	81.9 ± 6.7 (range: 57–97)	**0.032**
Female sex	72 (44%)	26 (32%)	46 (56%)	**0.003**
COPD	48 (29%)	23 (28%)	25 (30%)	0.864
Peripheral vascular disease	151 (93%)	74 (91%)	77 (94%)	0.565
Previous vascular surgery	21 (13%)	16 (20%)	5 (6%)	**0.010**
Coronary artery disease	109 (67%)	59 (73%)	50 (61%)	0.134
Previous CABG	37 (23%)	34 (42%)	3 (3.6%)	**<0.001**
Previous cardiac surgery	44 (27%)	41 (51%)	3 (3.6%)	**<0.001**
Previous coronary angioplasty/stenting	55 (34%)	28 (35%)	27 (33%)	0.869
Hypertension	121 (74%)	62 (76%)	59 (72%)	0.592
Chronic kidney failure	61 (37%)	35 (43%)	26 (32%)	0.147
Hemodialysis	11 (6.7%)	6 (7.4%)	5 (6%)	0.766
Previous stroke	5 (3.1%)	3 (3.7%)	2 (2.4%)	0.682
Diabetes on insulin	21 (13%)	12 (15%)	9 (11%)	0.493
Liver disease (CHILD B and C)	3 (1.8%)	1 (1.2%)	2 (2.4%)	1.000
Permanent pacemaker	25 (15%)	13 (16%)	12 (15%)	0.493
Critical preoperative state	5 (3.1%)	2 (2.5%)	3 (3.6%)	1.000
Calcified ascending aorta	22 (13%)	18 (22%)	4 (5%)	**0.001**
EuroSCORE-II (%)	4.2 (IQR: 2.8–7.9) Mean: 7.1 ± 8%)	5.0 (IQR:3.0–11.0 (Mean: 8.6 ± 7.9%)	3.9 (IQR: 2.5–5.4) (Mean: 5.6 ± 7.8%)	**0.005**

Data presented as mean ± SD, median (IQR) or N (%); *p*-value is a Fischer test for proportion, a Student’s *t*-test for age and a Mann–Whitney U test for EuroSCORE II. COPD = Chronic Obstructive Pulmonary Disease; CABG = Coronary Artery Bypass Grafting.

**Table 2:** Preoperative echocardiographic findings and CT scan measurements.

VARIABLES	Overall (*n* = 163)	TA-TAVR (*n* = 81)	TAO-TAVR (*n* = 82)	*p*-Value
Mean aortic valve gradient (mmHg)	38.1 ± 14.9	35.6 ± 13.2	40.4 ± 16.1	**0.045**
Mean aortic valve area (cm^2^)	0.7 ± 0.2	0.8 ± 0.2	0.7 ± 0.2	0.057
Mean left ventricular ejection fraction (%)	51.3 ± 13.6	49.1 ± 14.6	53.5 ± 12.3	**0.035**
Pulmonary hypertension (moderate-severe)	58 (37%)	30 (38%)	28 (35%)	0.742
Mean aortic annulus diameter at CT scan (mm)	24.1 ± 2.5	23.9 ± 2.1	24.3 ± 2.8	0.442
Mean aortic annulus diameter at TOE (mm)	23.3 ± 2.2	23.4 ± 2.2	23.1 ± 2.2	0.450

Data presented as mean ± SD or N (%). *p*-value is a Fischer test for pulmonary hypertension, a Student’s *t*-test otherwise. CT = Computed Tomography; TOE = Transoesophageal Echocardiogram.

**Table 3:** Procedural details.

VARIABLES	Overall (*n* = 163)	TA-TAVR (*n* = 81)	TAO-TAVR (*n* = 82)	*p*-Value
Valve-in-valve (degenerated bioprosthesis)	7 (4.3%)	6 (7.4%)	1 (1.2%)	0.064
Bailout TAV-in-TAV (malpositioning)	4 (6.3%)	3 (3.7%)	1 (1.2%)	0.367
*TAVR valve type:*				**<0.001**
Sapien-XT	20 (12.3%)	17 (21%)	3 (3.6%)	-
Sapien-3	69 (42.3%)	39 (48%)	30 (36.6%)	-
Sapien-3 Ultra	31 (19%)	21 (26%)	10 (12.2%)	-
Medtronic CoreValve	39 (24%)	0	39 (47.6%)	-
Boston Accurate	4 (2.4%)	4 (5%)	0	-
Mean TAVR valve size (mm)	26.5 ± 2.9	25.7 ± 2.3	27.2 ± 3.3	**0.009**
*TAVR valve size distribution:*				**0.003**
23 mm	41 (25.2%)	23 (28.4%)	18 (22%)	-
26 mm	66 (40.5%)	36 (44.4%)	30 (36.5%)	-
29 mm	42 (25.8%)	18 (22.2%)	24 (29.3%)	-
34 mm	10 (6.1%)	0	10 (12.2%)	-
M	2 (1.2%)	2 (2.5%)	0	-
L	2 (1.2%)	2 (2.5%)	0	-
Procedural time (min)	100 (IQR: 85–123)	97 (IQR: 882–118)	102 (IQR: 88–129)	0.133
Contrast injected (mL)	148.8 ± 101.4	125.2 ± 105.5	174.6 ± 90.7	**<0.001**

Data presented as mean ± SD, median (IQR) or N (%); *p*-value is a Fisher exact test for proportions and a Student’s *t*-test or a Mann–Whitney U test for continuous variables. TAV = Transcatheter Aortic Valve.

**Table 4:** Outcome at 30 days.

VARIABLES	Overall (*n* = 163)	TA-TAVR (*n* = 81)	TAO-TAVR (*n* = 82)	Difference (95% CI)	*p*-Value
Hospital mortality	10 (6%)	5 (6%)	5 (6%)	0% (−7 to 7)	1.000
*Cause of death:*					
Aortic annulus rupture	1 (0.6%)	0	1 (1.2%)		-
Ischemic bowel	2 (1.2%)	1 (1.2%)	1 (1.2%)		-
Pulmonary embolism	1 (0.6%)	1 (1.2%)	0		-
Cardiogenic shock	2 (1.2%)	1 (1.2%)	1 (1.2%)		-
Multiple organ failure	3 (1.8%)	2 (2.4%)	1 (1.2%)		-
Unwitnessed sudden death	1 (0.6%)	0	1 (1.2%)		-
Conversion to sternotomy (bleeding from the aorta)	1 (0.6%)	0	1 (1.2%)	0% (−3 to 1)	1.000
Re-exploration for bleeding	5 (3%)	2 (2.4%)	3 (3.6%)	−1.2 (−6.4 to 4.1)	1.000
Major vascular complication	4 (2.4%)	1 (1.2%)	3 (3.6%)	−3.6 (−8.9 to 1.6)	0.367
Major and life-threatening bleeding	5 (3%)	2 (2.4%)	3 (3.6%)	−1.2 (−6.4 to 4.1)	1.000
Extubation in the hybrid room	115 (70%)	55 (69%)	60 (73%)	−4.4% (−18 to 9)	0.605
Stroke/TIA	0	0	0	-	-
Postoperative acute kidney failure	12 (7.4%)	8 (9.9%)	4 (4.9%)	5.0% (−3 to 13)	0.247
Transitory dialysis	5 (3%)	3 (3.7%)	2 (2.4%)	1.3% (−4 to 6)	0.682
New permanent pacemaker implantation	15 (9%)	7 (8.6%)	8 (9.8%)	−1.1% (−10 to 7)	1.000
Intensive Care Unit stay (days)	1 (IQR: 1–2)	1 (IQR: 1–1)	1 (IQR: 1–1)		0.697
Hospital stay (days)	9 (IQR: 6–12)	10 (IQR: 7–13)	8 (IQR: 6–11)		**0.025**
Left ventricular ejection fraction at discharge (%)	52.1 ± 12.9	48.9 ± 13.5	55.3 ± 11.5		**0.002**
Mean aortic valve gradient at discharge (mmHg)	9.7 ± 4.2	9.7 ± 4.8	9.6 ± 3.5		0.830
Moderate-to-severe PVL	4 (2.4%)	2 (2.4%)	2 (2.4%)		1.000
None–mild PVL	159 (97.6%)	79 (97.5%)	80 (97.6%)		-
Moderate PVL	4 (2.4%)	2 (2.4%)	2 (2.4%)		-
Severe PVL	0	0	0		-

Data presented as mean ± SD, median (IQR) or N (%); *p*-value is a Fisher exact test for proportions and a Student’s *t*-test or a Mann–Whitney U test for continuous variables. TIA = Transitory Ischemic Attack; PVL = Paravalvular Leak.

## Data Availability

The data underlying this article cannot be shared publicly due to patient confidentiality. The data will be shared on reasonable request with the corresponding author.

## Abbreviations

TAVR	Transcatheter Aortic Valve Replacement
TF	Transfemoral
CT	Computed Tomography
TA	Transapical
TAO	Transaortic
PVL	Paravalvular Leak
VARC	Valve Academic Research Consortium
TIA	Transitory Ischemic Attack
